# Keeping an eye on Parkinson’s disease: color vision and outer retinal thickness as simple and non-invasive biomarkers

**DOI:** 10.1007/s00415-025-13080-6

**Published:** 2025-04-21

**Authors:** Jingjing Lin, David I. Finkelstein, Andrew J. Anderson, Pei Ying Lee, Bang V. Bui, Tissa Wijeratne, Jane E. Alty, Christine T. O. Nguyen

**Affiliations:** 1 Department of Optometry and Vision Sciences, The University of Melbourne, Melbourne, VIC Australia; 2 Florey Institute of Neuroscience and Mental Health, The University of Melbourne, Melbourne, VIC Australia; 3 Department of Neurology, Sunshine Hospital, Melbourne, VIC Australia; 4 Wicking Dementia Research and Education Centre, University of Tasmania, Hobart, TAS Australia; 5 Neurology Department, Royal Hobart Hospital, Hobart, Australia

**Keywords:** Neurodegenerative disorders, Parkinsonism, Visual impairment, Optical coherence tomography

## Abstract

Over the last two decades, visual symptoms and retinal changes in Parkinson’s disease (PD) have emerged as important biomarkers. Color vision deficiency, which begins in the outer retina, has been increasingly investigated, but a focused review of these papers has not recently been conducted. Similarly, thinning of the outer retina as measured using optical coherence tomography (OCT) holds potential as a screening marker for PD, particularly as these devices are already commonplace in community and hospital settings. Moreover, outer retinal thinning may be more specific for Parkinson’s disease as inner retinal changes also occur in more common neurodegenerative diseases like glaucoma and Alzheimer’s disease. This review summarizes contemporary evidence on two *outer retina* focused measures, color vision and outer retinal thickness, which can be readily quantified using non-invasive approaches and thus examines their potential as biomarkers for screening, detection, and progression in PD.

## Introduction

### The need for low-cost and scalable biomarkers

Parkinson’s disease (PD) is a fast-growing neurodegenerative disorder globally [1], and it is predicted that there will be over 12 million people living with PD (pwPD) worldwide by 2040 [2]. Clinical diagnosis of PD mainly relies on the presence of motor features, such as tremor, bradykinesia, and muscle rigidity [3]. However, studies have shown that by the time these cardinal symptoms present, substantial and irreversible neuronal loss (up to 70%) has already occurred in the substantia nigra [4, 5]. As such, there is a critical need for tools that help identify PD earlier in the disease course, as well as effectively track progression. Current brain-related biomarkers of PD are either costly (magnetic resonance imaging, positron emission tomography scans) or invasive; for example, the new alpha-synuclein seed amplification assays require cerebrospinal fluid or skin biopsy tests [7–9]. Thus, there remains a need for a scalable biomarker of PD that can be used at a population level, considering the large and escalating number of pwPD [6].

### Why should we look into the eyes?

Eye-related biomarkers have garnered interest in PD as visual abnormalities are common non-motor symptoms in the clinically diagnosed stage of PD [10–18]. Although subtle for a patient to notice, they can be readily quantifiable in the clinic. Importantly, there is evidence that they occur up to a decade before PD diagnosis, within the prodromal stage [19]. People with idiopathic rapid eye movement sleep behavior disorder (iRBD) are a prodromal group of great interest as they have a 70–90% risk of developing PD and related neurodegenerative disorders (i.e., dementia with Lewy Bodies, multiple system atrophy) [20–22]. However, iRBD is under-recognized and under-diagnosed in the community, partly related to poor awareness and inaccessible specialist sleep study tests [23, 24], and yet we know that early vision changes have been found in this group, too [25].

The mechanisms underlying visual symptoms in PD are an exciting area of research and include investigation of the two PD hallmarks, namely dopaminergic deficiency [26–28] and misfolded alpha-synuclein in the retina [29–32]. Overall, similarities between the retina and brain in terms of embryology, structure, and PD-related pathology [14, 33, 34] make the retina a logical and promising avenue as a site to search for early PD biomarkers. More specifically, there is a growing literature that the *outer retina* is altered in PD [35]. This includes converging evidence across functional studies, such as color vision which commences in the outer retina [36], electrophysiology which assays outer retinal activity in vivo [37], and retinal imaging studies including optical coherence tomography (OCT) [38–40] and OCT angiography [41–44] which return outer retinal neuronal structure and vascular structure respectively. This review will focus on *color vision and outer retinal OCT* given their greater accessibility and thus potential as implementable biomarkers in PD.

### Systematic review of the literature

To date, there is a lack of review papers that focus on the outer retina, and thus, the current manuscript specifically examines two tools that enable assessment of the outer retina, with the potential for widespread clinical implementation: color vision and outer retina OCT changes. We aimed to systematically review the literature to provide a narrative summary of the current understanding of color vision impairment in PD and explore opportunities for outer retina OCT parameters to serve as biomarkers for the detection of PD, including in its prodromal stage as well as its progression.

This narrative review included a literature search conducted in the PubMed database, with a search term of (“Parkinson” [All Fields] OR “rapid eye movement sleep behavior disorder”) AND (“optical coherence tomography"OR “OCT” [All Fields]), as well as (“Parkinson” [All Fields] OR “rapid eye movement sleep behavior disorder”) AND (“colo(u)r vision” OR “colo(u)r discrimination” OR “colo(u)r vision deficiency” [All Fields]). First, research articles with their abstract and result section mentioning any of the outer retinal layers (including outer plexiform layer (OPL), outer nuclear layer (ONL), photoreceptors layer (PRL), and retinal pigment epithelium (RPE)) or colo(u)r vision information were screened. Second, the full text was reviewed to determine whether color vision and outer retinal OCT were detailed in the method section for inclusion. Third, the reference articles from all screened studies were checked. Cohen’s d Effect Size [45] was calculated to measure the statistical power based on data (numbers of samples, the mean value, and standard deviation of compared parameters) provided in research articles.

## Color vision

### What exactly is color vision?

Color vision is the ability to distinguish chromatic or “colored” objects [46] and involves both retinal and cortical components of the visual pathway. In the retina, three types of cone photoreceptors are responsible for color vision: S cones, M cones, and L cones, reflecting preferential sensitivity to short, medium, and long wavelengths of light, respectively. Lights with different spectral distributions can produce differences in the relative activation of these three cone classes, with this information then processed through bipolar cells in the middle retina, laterally communicated through amacrine cells, then transmitted to ganglion cells in the innermost retina, and then to the brain. In the brain, both achromatic (luminance) and chromatic (color) signals pass through the lateral geniculate nucleus in the thalamus and onward to the primary visual cortex. Visual information is streamed into three pathways: magnocellular (luminance), parvocellular (red–green), and koniocellular (blue–yellow). Therefore, impairment at any stage in the visual pathway can lead to aberrant color discrimination [46].

Congenital color vision deficiency is relatively common in the population (8% in males and 0.5% in females) and largely occurs on the red–green axis [47]. On the other hand, acquired color vision deficiencies, including those caused by acquired retinal disease and by exposure to environmental/toxins may involve the blue–yellow axis [48].

### How is color vision currently assessed?

**Fig. 1:**
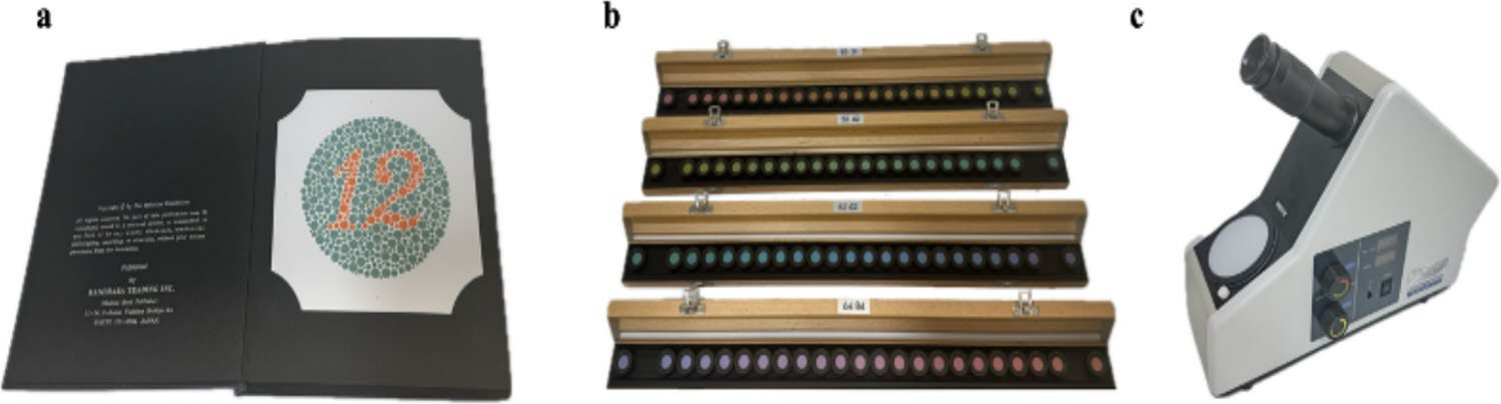
Common clinical color vision tests. **a** Ishihara test (24-plate edition 2011, Kanehara Trading Inc., Tokyo, Japan); **b** Farnsworth–Munsell 100-Hue test (FM 100-Hue, X-Rite, Grand Rapids, MI, USA); **c** Anomaloscope test (OT-II, Neitz Instruments Co., Ltd., Tokyo, Japan)

Common clinical tests of color vision include pseudoisochromatic plates (e.g., Ishihara test, Fig. 1a), hue arrangement tests (e.g., Farnsworth–Munsell 100-Hue test, Fig. 1b), and color matching tests (e.g., anomaloscope, Fig. 1c). Different kinds of tests serve different purposes (i.e., screening or gold standard assessment; congenital versus acquired color vision deficiencies) in clinical practice and research. The anomaloscope is the gold standard color vision test and involves matching a test color with two different colored lights but is mostly utilized for research purposes due to its expense and expertise to operate (Fig. 1c). On the other hand, the Ishihara test is widely used in clinic, especially for screening of congenital red–green color vision defects [49], and requires discerning numbers formed by dots within a field of dots in a different color (Fig. 1a). In the PD research literature, the FM- 100 Hue test is the most commonly used color vision test due to its high accuracy and moderate accessibility. The test involves arranging 85 colored caps in order of hue, with higher error scores indicating worse performance (Fig. 1b). Modern computerized tests of color vision enable tailored assessment of multiple aspects of color vision and with the digital age facilitate the potential for widespread implementation.

### Color vision deficiency in Parkinson’s disease

**Fig. 2:**
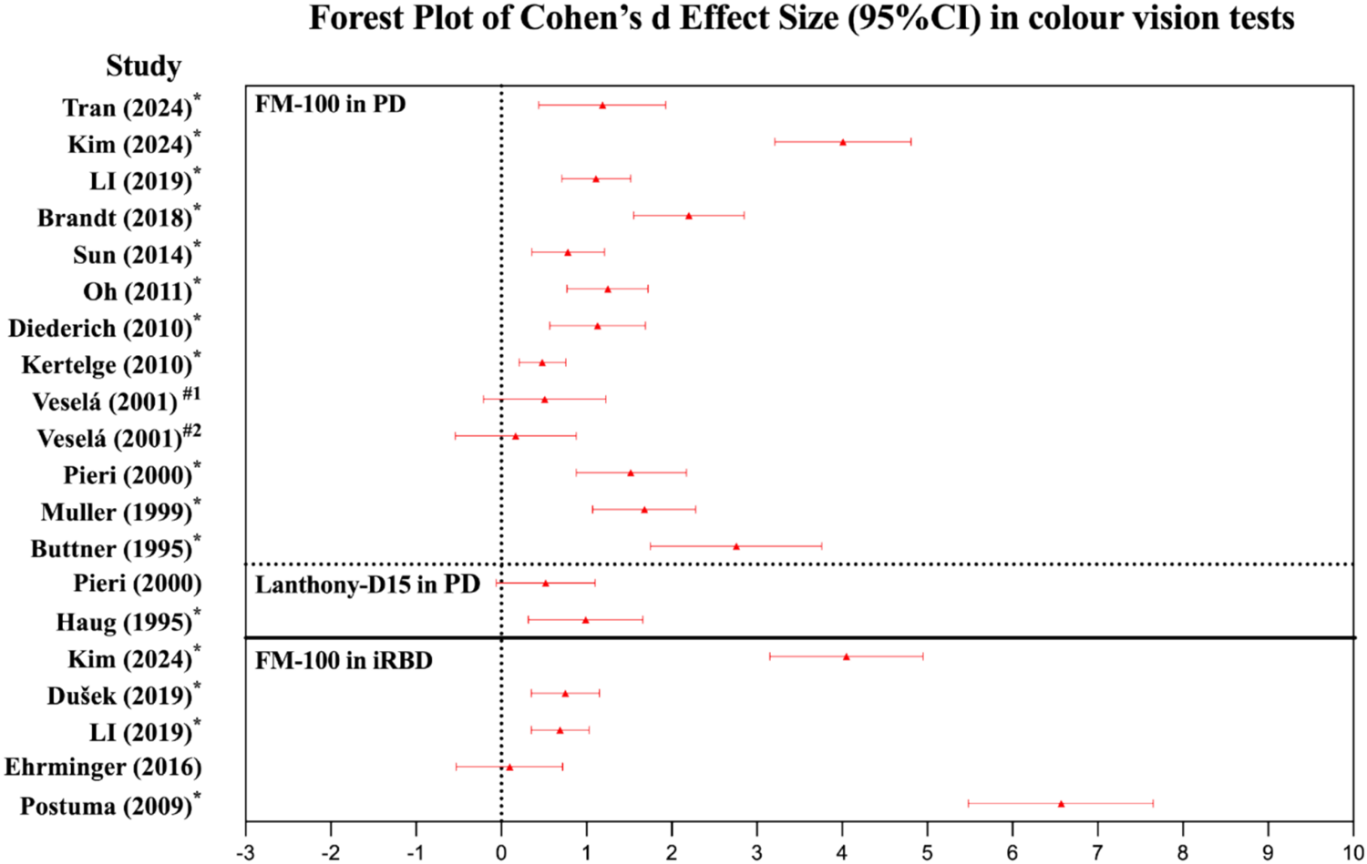
Forest plot showing Cohen’s d effect size (95% CI) on color vision discrimination tests. Each study is represented by a dot (mean Cohen’s d) with error bars (95% CI). Positive values suggest higher error scores in the PD (or iRBD) group compared to healthy controls. Asterisks (*) indicate significant differences between groups; ^#1^ and ^#2^ represent the first and the second FM- 100 test, respectively; CI, confidence interval; PD, Parkinson’s disease; iRBD, idiopathic rapid eye movement sleep behavior disorder; FM- 100, Farnsworth-Munsell 100-Hue test; Lanthony-D15, Lanthony Desaturated 15-Hue Test

As shown in Table 1 and Fig. 2, color vision deficiency has been evaluated in multiple cross-sectional studies of people with untreated PD who are drug-naive to dopaminergic medications [50, 51], pwPD treated with dopaminergic drugs [35, 52–56], as well as in longitudinal studies of PD [57–59]. Evidence from most studies suggests that deficits in color vision discrimination could differentiate pwPD from age-matched control cohorts, with significantly higher error scores in FM- 100 or Lanthony Desaturated 15-Hue Test (a modified version of the Farnsworth-D15 with lower saturation) in pwPD compared with healthy controls [35, 50, 52–56, 60–62], although one study found no difference [63]. Encouragingly, 83% (10/12) of studies (Fig. 2) identified significantly impaired color vision in PD and exhibited high Cohen’s d Effect size ranging from ~ − 0.6 to 4.9 (including 95%CI) regardless of cohort, age, sex, disease duration, disease severity, treatment, and color vision test settings (e.g., lighting conditions). One study found that 30 pwPD (mean age: 65.6 years old) and 30 age-matched healthy controls could be differentiated with both a specificity and sensitivity of 0.67 using a cut-off FM- 100 total error score of 115.2 [54].

**Table 1:** Color vision deficiency among people with PD and iRBD

Paper	Numbers of participants		Mean age (years)		Mean Hoehn and Yahr scale	Mean Disease duration (year)	Levodopa therapy (yes/no)	Color vision test	Findings (total error score (TES), AU, Mean ± SD)		
	PD (iRBD)	HC	PD (iRBD)	HC					PD (iRBD)	HC	*P* value
Color vision deficiency in PD											
Tran et al. (2024) [35]	16	19	61	57	–	5.0	Yes	FM- 100	123.5 ± 73.4	52.7 ± 45.3	< 0.01
Kim et al. (2024) [60]	43	32	68.8	71.1	–	6.0	–	FM- 100	225.9 ± 11.9	175.2 ± 13.6	0.018
Li et al. (2019) [66]	52	79	64.2	66.7	1.7	3.7	–	FM- 100	149.2 ± 63.5 (*N* = 46)	90.8 ± 43.6 (*N* = 69)	< 0.05
Brandt et al. (2018) [56]	30	34	70	60	1.8	6.0	Yes	FM- 100	327.0 ± 200.0	18.0 ± 9.0 (N = 31)	0.0001
Satue et al. (2017) [59]	40	–	74.6	–	2.9	18.4	Treat-free: 9%	Farnsworth-D15 and Lanthony-D15	Over 5-year, only Lanthony color confusion Index increased from 1.7 ± 0.6 to 2.2 ± 0.5 (*P* = 0.016)		
Sun et al. (2014) [61]	45	50	65.4	65.5	2.2	8.7	Yes	FM- 100	135.8 ± 84.8	84.3 ± 41.7	0.0001
Oh et al. (2011) [53]	54	34	69.0	65.4	1.8	4.3	Yes	FM- 100	182.6 ± 96.3	82.0 ± 45.6	0.001
Diederich et al. (2010) [54]	30	30	65.6	67.7	–	2.2	Yes	FM- 100	144.4 ± 59.8	86.5 ± 40.8	< 0.001
Kertelge et al. (2010) [67]	100	110	63.7	58.7	2.5	8.4	–	FM- 100	134.8 ± 92.8 (*N* = 98)^*^	97.2 ± 61.1 (*N* = 109)^*^	0.005
Silva et al. (2005) [68]	30	32	61.1	57.9	1.9	4.6	Treat-free: 33%	Computer-controlled test	PD participants showed impaired color vision in the red–green axis (both *P* < 0.01), and a trend that did not reach significance in the blue–yellow axis (*P* = 0.0591)		
Diederich et al. (2002) [58]	28	–	65.2	–	–	13.5	Yes	FM- 100 and Lanthony-D15	Over 19.8 ± 2.8 months, TES in FM- 100 increased from 99.1 ± 76.6 to 126.5 ± 103.5 (*P* = 0.02), while TES in Lanthony-D15 increased from 7.0 ± 9.3 to 6.4 ± 11.4 (*P* = 0.38)		
Muller et al. (2002) [57]	18	–	61.4	–	–	–	Yes	FM- 100	Over 3 years, TES increased from 69.7 ± 32.2 to 134.1 ± 85 (*P* = 0.002)		
Veselá et al. (2001) [63]	14	20	55.4	51.2	1.4	2.3	No	FM- 100	First trial: 49.1 ± 37	37.9 ± 25	< 0.528
									Second trial: 36.1 ± 31	31.4 ± 25	< 0.806
Pieri et al. (2000) [62]	21	30	69.9	67.9	2.6	9.6	Yes	Lanthony-D15 FM- 100	6.4 ± 8.9 121.7 ± 70	2.9 ± 4.8 37.7 ± 42	0.18 0.0001
Muller et al. (1999) [50]	30	30	58.7	57.6	2.0	–	No	FM- 100	79.4 ± 50.6	18.4 ± 9.6	0.0001
Haug et al. (1995) [55]	26	17	64.5	62.6	2.2	–	Yes	Lanthony-D15	10.5 ± 7.3	4.2 ± 4.1	0.001
Buttner et al. (1995) [51]	16	16	58.0	57.8	1.5	1.6	No	FM- 100	64.6 ± 24.0	16.0 ± 6.8	0.0001
Price et al. (1992) [52]	35	26	63	60	2.7	–	Yes	FM- 100	238.3	73.1	0.0001
Color vision deficiency in iRBD											
Kim et al. (2024) [60]	28	32	73.9	71.1	–	8.4	–	FM- 100	232.6 ± 14.8	175.2 ± 13.6	0.016
Dušek et al. (2019) [69]	74	39	67.5	65.2	–	6.5	–	FM- 100	102.3 ± 78.3	52.4 ± 34.2	< 0.0001
Li et al. (2019) [66]	83	79	67.9	66.7	–	7.3	–	FM- 100	125.6 ± 56.5 (*N* = 73)	90.8 ± 43.6 (*N* = 69)	< 0.05
Ehrminger et al. (2016) [70]	21	21	67.4	67.6	–	5.9	–	FM- 100	127.4 ± 63	120.5 ± 78.9	0.76
Postuma et al. (2009) [64]	68	36	68.0	65.8	–	9.3	–	FM- 100	174.6 ± 12.5 (*N* = 57)^*^	94.5 ± 11.6 (*N* = 30)^*^	< 0.001

*PD* Parkinson’s disease; *HC* Healthy controls; *FM- 100* Farnsworth-Munsell 100-Hue test; *Farnsworth-D15* Farnsworth–Munsell Desaturated 15-Hue Test (a simplified version of FM- 100); *Lanthony-D15* Lanthony Desaturated 15-Hue Test (a modified version of the Farnsworth-D15 with lower saturation); *AU* Arbitrary Units; *SD* standard deviation; *iRBD* idiopathic rapid eye movement sleep behavior disorder

^*^Exact number for participants who performed the FM- 100

### Color vision deficiency in prodromal Parkinson’s disease

Postuma and colleagues extended previous work in the PD field and examined color discrimination in prodromal PD; participants with iRBD [19, 20, 64, 65]. The initial two cross-sectional studies from Postuma et al. [64, 65] demonstrated a significant color vision impairment in an iRBD cohort with the FM- 100 test (57 polysomnography-confirmed RBD vs 30 age- and sex-matched healthy controls, *P* < 0.001). In 2019, they reported data collected from their longitudinal study (154 iRBD participants and 102 healthy controls, 2004–2016) and used a mixed effect model to predict that the first presentation of impaired color vision can occur 12.8 years before iRBD converted to neurodegenerative diseases such as PD, dementia with Lewy bodies, and multiple system atrophy [19]. Furthermore, with a cut-off error score in FM- 100 more than 153, the largest area under the receiver-operating characteristic curve (AUROC) for separating iRBD phenoconverters from controls based on FM- 100 was 0.71 at the year of phenoconversion, with a sensitivity of 0.70 and a specificity of 0.61; FM- 100 testing also gave an AUROC of 0.65 six years prior to phenoconversion, with a sensitivity of 0.56 and a specificity of 0.61. In comparison, quantitative measures of cardinal motor symptoms started 7–11 years prior to formal PD diagnosis [19]. Postuma et al. [20] also conducted an international multi-center study with the largest iRBD cohort to date of 1280 polysomnography-proven participants with a mean age of 66.3 years old (82.5% male), of which 240 participants completed the FM- 100. Importantly, the rate of iRBD participants phenoconverting to PD and related disorders was 69% higher in those with color vision abnormalities (hazard ratio = 1.69, 95% CI 1.01–2.78).

Interestingly, previous iRBD studies (Table 1) presented similar findings to PD research, as most studies (4/5) showed that iRBD participants had a significant increase in FM- 100 scores compared to healthy controls, with one relatively smaller study (21 iRBD participants vs 21 healthy controls) reporting no change and details regarding the experimental procedure were lacking (i.e., lighting conditions). The Cohen’s d Effect size ranged from − 0.53–7.65 (including 95%CI), and the mean value was high at 4.1 for Kim et al.’s study [60] and 6.7 for one of the studies from Postuma et al. [64] (Fig. 2). Of note, all participants from both studies had a gold standard (polysomnography-confirmed) of their iRBD. To avoid overlap in participants, the study selected from Postuma’s team was the latest and largest iRBD color vision cross-sectional manuscript. Whether the trend for greater effect size in studies that examine polysomnography-confirmed iRBD compared with studies that test diagnosed PD cases is a reflection of only a subset of pwPD also having iRBD; whether people with iRBD and color vision dysfunction are more likely to convert to other non-PD degenerative disorders (such as dementia with Lewy bodies, and multiple system atrophy) requires further investigation [19].

### The underlying pathology of color vision loss in Parkinson’s disease

Previous studies have suggested that color vision loss in PD is due to dopamine deficiency affecting the visual system potentially at retinal and cortical sites. Indirect evidence provides support for this idea, with two studies reporting that levodopa therapy could improve color vision [61, 71]. This proposed pathological mechanism aligns with the loss of dopaminergic cells in the substantia nigra [72], as well as decreased dopamine levels [26] and impaired dopaminergic cell function [28] in the retina [27]. In the human retina, dopaminergic cells are mainly amacrine cells [28], the impairment of which can alter signal processing in the visual pathway [73]. Interestingly, the dopaminergic subclass of amacrine cells is the only amacrine cells that have processes that extend to the outer retina [74]. In a previous study by our group [35], pwPD demonstrated greater color vision impairment on FM- 100, decreased outer retinal photoreceptor function on electrophysiology, and degeneration of outer retinal photoreceptor cell nuclei measured as a thinner retinal ONL on OCT scans compared with age-matched healthy controls. Given that the macula ONL consists largely of cones [75], these data identify a potential association with the observed color vision loss.

In 1995, Haug et al. [55] found significantly increased thresholds responding to stimuli on the blue–yellow compared to the red–green axis in PD participants and speculated that the more vulnerable S cones compared to M cones and L cones in the retina contributed to the result. Sun et al. [61] reported similar findings. However, some researchers failed to detect differences in the blue–yellow axis in PD [58, 63, 76]. In contrast to Haug and co-workers, Silva et al. [68] performed detailed eye exams in the participant inclusion to mitigate the influence of confounding factors such as age-related ocular changes and found that deficits in the blue–yellow axis were not as marked as deficits in the red–green axis, which were considered distinct from age and as disease-specific. Nevertheless, a former study conducted by Regan et al. [77] used the same color vision test but detected no difference in color vision on any axis between pwPD and healthy controls. The lack of differences might be due to these participants with red–green color deficiencies being pre-excluded with the Ishihara test, an effort to exclude congenital color vision loss but may have also excluded PD-related color deficiencies.

Collectively, the exact contributions of retinal and cortical components, as well as retinal cells and visual pathways to color vision deficit in PD require further investigation. Future studies are needed to determine whether there is a consistent pattern of color vision dysfunction in pwPD and whether this varies across different subtypes of PD and alters with disease course.

### Linking color vision to the disease stage of Parkinson’s disease

Previous literature has examined the severity of PD and how it is related to color vision deficiency. Currently, both the Hoehn and Yahr stage [78] and the Movement Disorder Society-Unified Parkinson’s Disease Rating Scale (MDS-UPDRS) [79] are used to assess the severity of PD, but there is variation in the consistency of rating across clinics [80]. The Hoehn and Yahr staging is a categorical scale that mostly focuses on motor symptoms, while the MDS-UPDRS measures self-reported information and examination results in 4 separate parts, including “non-motor experiences of daily living”, “motor experiences of daily living”, “motor examination”, and “motor complications”. However, neither of these scales includes items directly related to assessing color vision impairment, or the relationship between color vision deficiency and the severity of PD. A number of studies have reported that higher Hoehn and Yahr stage [53, 66] or higher whole UPDRS [53, 57, 58, 81] are associated with worse performance in FM- 100. It is speculated that the deterioration in color vision is correlated with other visual deficits, such as reduced contrast sensitivity (an indicator of how well people can detect objects within a background) [62], stereopsis [61] and oculomotor[81] impairment (which are driven by how well the two eyes work together), and even visual hallucinations [82], that can indirectly impact the motor experiences in daily life [57, 58]. Conversely, other researchers have demonstrated no association between FM- 100 error score and PD stage [51, 60, 83, 84]. Interestingly, Muller et al. [84] found that motor impairment tested by peg insertion abilities was significantly associated with FM- 100 error score, while no association was found between the total UPDRS score and FM- 100 error score. Whether color vision discrimination could be used to stratify different stages of PD needs to be further investigated with larger cohorts and longitudinal observation.

### Related disturbing factors in color vision assessment of Parkinson’s disease

Age is considered the most important confounder of potential ocular PD biomarkers, as healthy aging can cause changes in the eye and increase disease risk [34, 36, 85]. Performance on color vision tests, especially on the blue–yellow axis, can also be affected by aging, which arises primarily due to lenticular senescence and preferentially reduces the transmission of blue light to the retina [86]. However, age-related deficits in color discrimination have not been consistently reported, as some researchers have found a positive significant correlation between color discrimination thresholds and age [61, 77], which contradicts others that failed to find significant correlations [62, 81]. In previous cross-sectional PD studies, most researchers have pre-excluded participants with age-related ophthalmologic changes (such as cataracts, glaucoma, and age-related maculopathy) to mitigate the influence of age in the eye [51, 60, 83, 84].

Another critical issue raised by researchers employing color vision tests in pwPD is the testing method. Despite the FM- 100 exhibiting high test–retest errors [87], it also requires certain executive ability when participants arrange small colored caps. Therefore, some researchers have suggested that altered motor capacity [81, 84] in pwPD could influence FM- 100 results, and thus, this test might not precisely reflect color vision discrimination deficits in pwPD [64, 68, 77, 81, 83, 84]. Additionally, impairment of saccadic eye movement [88] and convergence ability [89] in pwPD might also contribute to increased errors. Moreover, the performance of FM- 100 can be significantly influenced by cognitive dysfunction [64, 83]. PwPD with cognitive decline may perform worse with the FM- 100 because this test also requires executive abilities such as working memory and planning, which can be impaired in pwPD [90].

Consequently, there has been an interest in pseudoisochromatic tests (such as the Ishihara test, Fig. 1a), which are generally easier to understand and perform in clinical practice compared with color cap arrangement tests (Fig. 1b) [91]. However, results with pseudoisochromatic tests have been variable. Kupersmith et al. [92] administered the Ishihara test to 30 PD participants with a mean age of 52 years and found no color abnormalities, which was consistent with the previous results in similar sample sizes of PD participants (from 19 to 30) [58, 93, 94]. In contrast, Suciu et al. [95] performed the Ishihara test and detected an abnormality rate of 33% (10/30) in pwPD with a mean age of 68 years old. Additionally, Bradvica et al. [96] recruited 59 participants and revealed a high specificity of 88.2% and a sensitivity of 55.9% in distinguishing early pwPD (disease duration within 1 year of diagnosis) from age-matched healthy controls with the Ishihara test. However, it is important to note that positive results with the Ishihara test could also include congenital color vision deficiencies, and this test lacks evaluation of the blue–yellow axis. Particularly focusing on tests for blue–yellow deficits, Birch et al. [76] tested 44 PD participants (mean age was 68.5 years) and 40 age-matched normal controls on a range of clinical plates and color arrangement tests, including the City University Tritan plates, SPP2 (Tritan designs), Lanthony Tritan Album, Sahlgren’s Saturation Test, T16 and Adams D15. They showed that only the Adams D15, which was a color arrangement test, reached a high sensitivity of 95% and a specificity of 57.5% [76]. A major limitation of using conventional plate tests, compared to arrangement tests, is that these tests produce categorical pass/fail results instead of quantitative variables, and as such, small changes can be missed which may contribute to the differences seen in the different tritan tests employed by Birch et al. [76].

Some researchers have recommended using computerized color vision test devices to address the above limitations of FM- 100 [68]. Previous studies have employed customized lab-based examinations, and some variation exists between studies. Haug et al. [55, 97] used a computer-controlled system the ‘Moorfield Vision System’ and found a significant elevation of color thresholds along the blue–yellow axis. Others employed a ‘Cambridge Research Systems’ platform and revealed a preferential red–green loss [68], but another group failed to find a significant loss of color vision in either axis using the same platform with different experimental parameters [77]. Current commercially available computerized color vision test devices, such as the Cone Contrast Test [98, 99] and the Colour Assessment and Diagnosis test [100, 101], have not yet been examined in PD, but further research may aid in determining their utility for PD in terms of screening and monitoring.

Previous evidence has repeatedly shown that both PD and prodromal PD (iRBD cohorts) exhibited impaired color vision assessed by the FM- 100 test. However, the FM- 100 is not a common approach in standard clinical eyecare practice [49] and is used predominantly in specialized color vision clinics under specific lighting conditions. Given these challenges with current color vision tests, there would still be utility in the field for a simple-to-administer robust color vision test that could be implemented on a population scale for PD screening.

## Outer retinal thickness

### Outer retinal OCT: a scalable biomarker

**Fig. 3:**
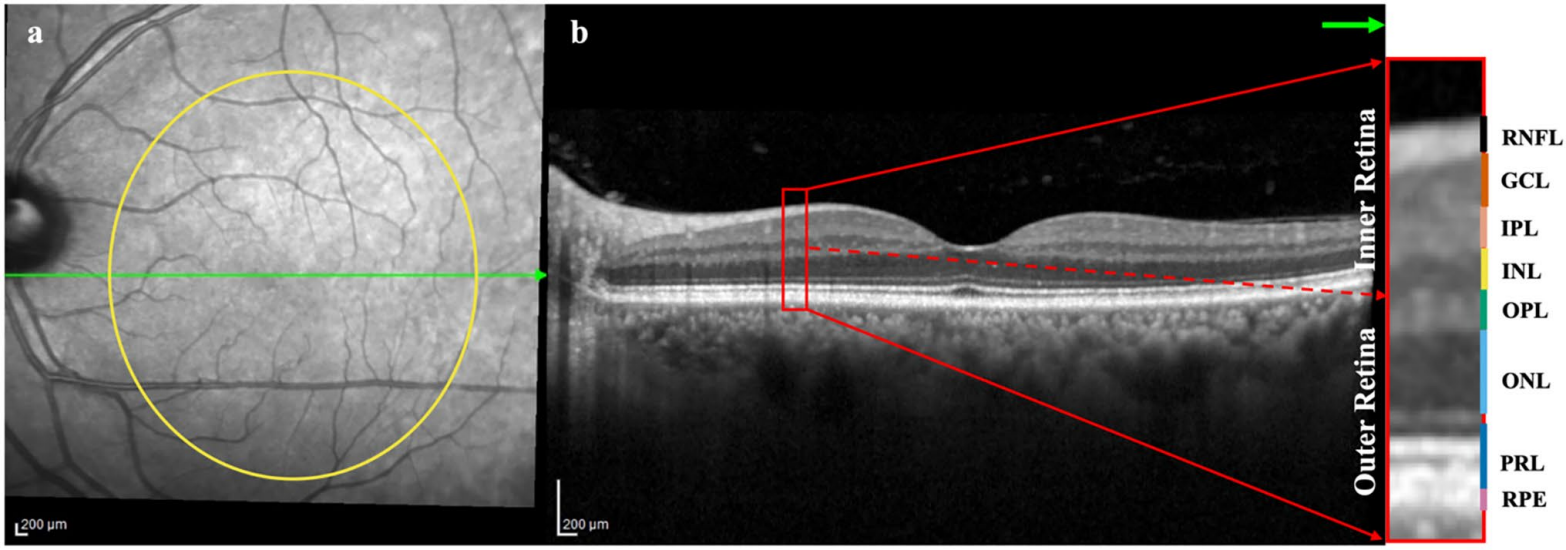
A representative single-line OCT scan. **a** An enface image of the posterior retina. The yellow circle represents the macular region of the retina, 6 mm X 6 mm in size. The green line indicates the path of a single macular OCT line scan displayed in panel (**b**). **b** A cross-sectional (B-scan) OCT image of the posterior retina in the horizontal meridian. The cross-sectional OCT image returns retinal layers including the **inner retina**: RNFL (black layer), retinal nerve fiber layer; GCL (orange layer), ganglion cell layer; IPL (beige layer), inner plexiform layer; INL (yellow layer), inner nuclear layer; and the **outer retina**: OPL (green layer), outer plexiform layer; ONL (light-blue layer), outer nuclear layer; PRL (dark-blue layer), photoreceptors (rods and cones) layer; RPE (pink layer), retinal pigment epithelium. The macular region is a key area which is responsible for visual acuity and color vision. There are three main types of neurons in the retina located in different layers: 1. photoreceptor cells (dendrites PRL, somas in ONL, and axons in OPL); 2. bipolar cells (dendrites in OPL, somas in INL, and axons in IPL); 3. ganglion cells (dendrites in IPL, somas in GCL, and axons in RNFL), and visual signals are transmitted from outermost to innermost layer of the retina

As shown in Fig. 3, the retina is a well-organized tissue with multiple layers, and its structure can be assayed in vivo using OCT, a widespread, non-invasive, and rapid method. The macula area of the retina contains a high density of cones, which contribute to color vision and the ability to see fine details. A reduction of retinal layers in thickness or volume quantified using OCT can localize neurodegeneration or the loss of neural tissue to a particular sub-layer of cells. In contrast, the thickening of layers can reflect swelling, gliosis, as well as accumulation of deposits.

OCT is a high-resolution in vivo imaging method that allows the assessment of retinal structure in both clinical (including community and hospital) and research settings. OCT employs low-coherence light to measure backscattering properties, which differ between nuclei and dendritic layers of neural tissue in the retina [102, 103]. In the past 20 years, OCT technology has rapidly advanced, and commercial devices can now reveal retinal structures with high resolution (down to 3 um [104]), excellent repeatability, and in a matter of seconds due to high scanning speed [105, 106]. Built-in automatic segmentation and analysis algorithms reliably quantify ten individual retinal layers (illustrated in Fig. 3). Common automated analysis outputs focus on the inner retina, as nerve fiber layer thinning is an important indicator of common eye diseases such as glaucoma. Similarly, OCT application in PD has also focused on inner retinal OCT changes in PD [107–112]. However, inner retinal thinning can occur in a range of conditions, including Alzheimer’s disease, multiple sclerosis, and traumatic brain injury. The emergence of imaging and analysis approaches to focus our attention on the outer retina increases the likelihood of improved specificity for PD.

### Structural OCT changes in the outer retina of people with Parkinson’s disease

In recent PD studies, OCT has been used to quantify retinal layer thicknesses in pwPD [39]. Inzelberg et al. [113] in 2004 was the first to examine OCT in pwPD and found a significant decrease in retinal nerve fiber layer (RNFL, axons of ganglion cells) thickness in pwPD compared with age-matched controls. Since then, many studies have assessed differences in inner retinal ganglion cell-related layers, including the RNFL, ganglion cell layer, and inner plexiform layer between pwPD and healthy participants [107–112]. A recent meta-analysis [110] of 36 studies found that pwPD demonstrated a significant thinning in inner retinal ganglion cell-related layers (i.e., RNFL, macular ganglion cell complex), as well as overall thickness at the macula (macular volume and macula thickness) compared with age-matched healthy controls. Fewer studies have examined outer retinal changes in pwPD, and a recent review that focuses on these is lacking.

**Fig. 4:**
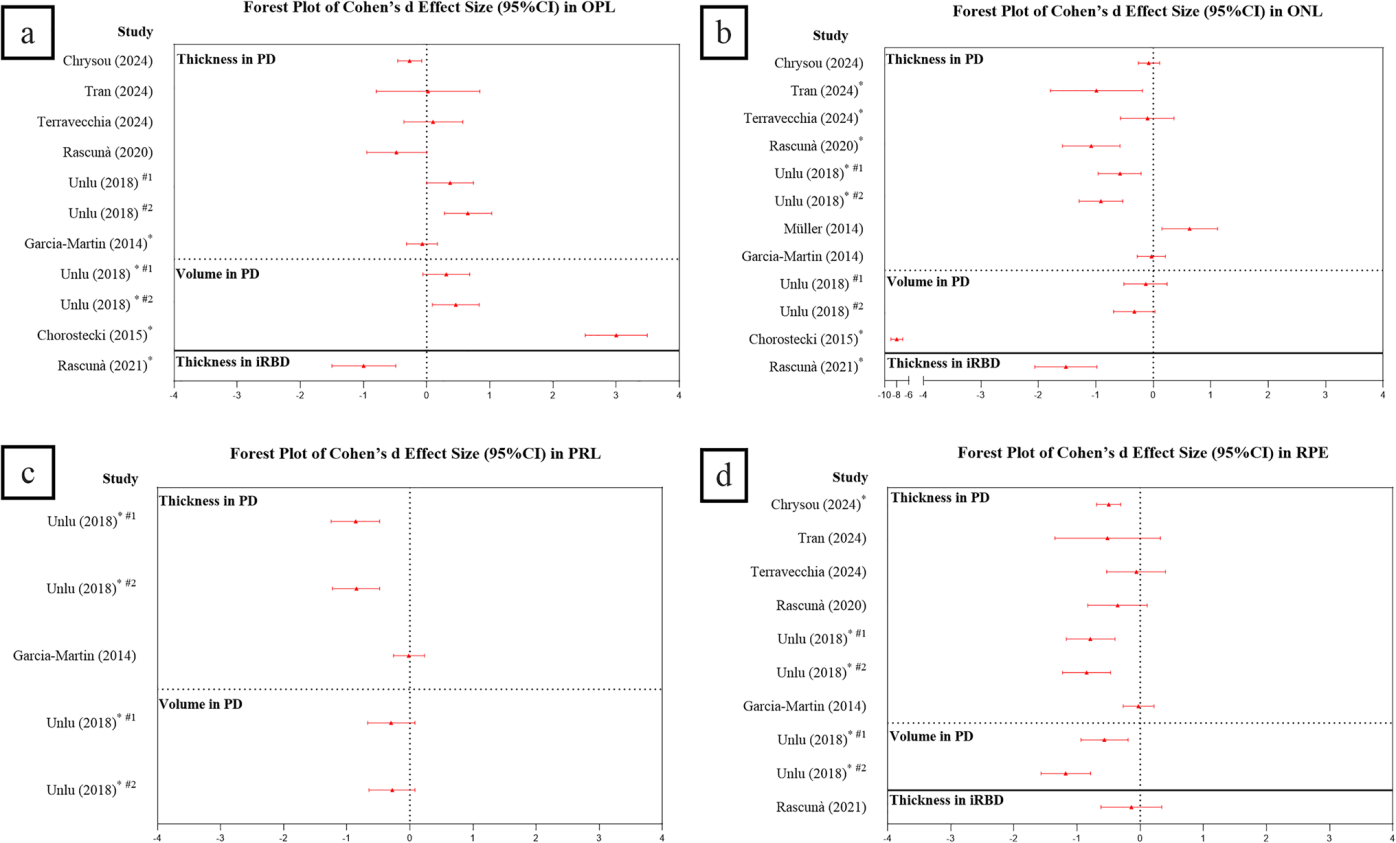
Forest plot showing Cohen’s d effect size (95% CI) in studies reporting the thickness of individual outer retinal layers [**a** OPL, **b** ONL, **c** PRL, and **d** RPE]. Each study is represented by a dot (mean Cohen’s d) with error bars (95% CI). Positive values represent thickening of the layers in the PD or iRBD group compared to healthy controls, while negative values suggest thinning of the layer. Asterisks (*) indicate significant differences between groups; ^#1^ and ^#2^ represent unilateral PD and bilateral PD, respectively; *CI* Confidence interval; *PD* Parkinson’s disease; *iRBD* idiopathic rapid eye movement sleep behavior disorder

Table 2 summarizes the current outer retinal OCT literature including studies that examine outer retinal changes in a single outer retinal layer and combined outer retinal layers. These are ordered from the most to least recent publications. The more recent papers published 2014–2024 employ the current gold standard three-dimensional OCT scans which evaluate thickness across a wide region of the retina (Table 2A). Older studies utilize a single-line scan reflecting the availability of the technology at the time (2012–2014). Interestingly, none of the older studies employing single horizontal line scans in PD demonstrate a change in the outer retina. In contrast more comprehensive recent studies employing volume scans more consistently report changes. These changes are visualized in Fig. 4, where data from the four commonly reported outer retinal OCT metrics have been expressed as a Cohen’s d effect size. More specifically, the OPL (outer plexiform layer, consisting of photoreceptor dendrites that connect to the downstream bipolar cell dendrites), ONL (outer nuclear layer that represents photoreceptor cell bodies), PRL (photoreceptor layer which includes photoreceptor inner and outer segments), and RPE (retinal pigment epithelium, support cells for outer retinal photoreceptors) are plotted for 3D OCT volume scans. Volume scans were analyzed to return average “thickness” or total “volume”.

**Table 2:** Outer retinal thickness in OCT among people with PD and iRBD

Papers	Numbers of participants (eyes)		Mean age (years)		Mean Hoehn & Yahr scale	Mean disease duration (year)	OCT Scanning			Region of interest	Findings in outer retinal layers			
	PD	HC	PD	HC			Machine	Scan mode	Segmentation method		OPL	ONL	PRL	RPE
**A.** Volume OCT scans														
Chrysou et al. (2024) [104]	121 (242)	100 (200)	65.1	62.7	2	< 0.25	Canon HS- 100 OCT	Volumetric macular region	Automatic	Mean thickness of the macular area (6 mm X 6 mm)	↔	↔	ISOS: ↔ OSL: ↔ OPR: ↔	↓
Tran et al. (2024) [35]	16 (11)	21 (15)	61	57	NA	5	Spectralis OCT	Volumetric macular region	Automatic	Mean thickness of the macular area (6 mm X 6 mm)	↔	↓	NA	↔
Terravecchia et al. (2024) [114]	21 (41)	17 (33)	61.5	65.1	NA	2.3	Cirrus HD-OCT	Volumetric macular region	Semi-automated	Mean thickness of the macular area (6 mm X 6 mm)	↔	↓	NA	↔
Mello et al. (2022) [37]	30 (41)	19 (38)	58.5	52.5	2.1	9.1	Spectralis OCT	Volumetric macular region	Semi-automated	Mean thickness of the macular area (individual sectors in 6 mm X 6 mm ETDRS)	↔	↔	↔	↔
Zhang et al. (2021) [115]	52 (100)	100 (200)	57.9	56.6	1.5	2.5	VG200 (SS-OCT)	Volumetric macular region	Semi-automated	Mean thickness of the macular area (individual sectors in 6 mm X 6 mm ETDRS)	ORL: ↓			
Rascunà et al. (2021) [25]	19 (37)** (iRBD)**	17 (33)	58.8	65.1	NA	NA	Cirrus HD-OCT	Volumetric macular region	Automatic	Mean thickness of the macular area (6 mm X 6 mm)	↓	↓	NA	↔
Rascunà et al. (2020) [116]	21 (41)	17 (33)	61.5	65.1	1.9	2.3	Cirrus HD-OCT	Volumetric macular region	Automatic	Mean thickness of the macular area (6 mm X 6 mm)	↔	↓	NA	↔
Unlu et al. (2018) [117]	58 (116)	30 (60)	60.5	60.2	2.1	7.1	Spectralis OCT	Volumetric macular region	Automatic	Mean thickness and mean volume of the macular area (6 mm X 6 mm)	Thickness			
											↔	↓	↓	↓
											Volume			
											↑	↔	↓	↓
Uchida et al. (2018) [118, 119]	22 (22)	36 (36)	62.9	65.1	NA	NA	Cirrus HD-OCT	Volumetric macular region	Automatic	Mean thickness at the fovea central, temporally and nasally 1 mm from the fovea. Mean *volume* of the macular area (6 mm X 6 mm)	Both thickness and volume			
											NA	ONL+ PRL: ↔		NA
Mailankody et al. (2015) [120]	30 (60)	30 (60)	53.4	53.5	1.7	5.3	Spectralis OCT	Radial macular scans	Automatic	Mean thickness at 0.5 mm and 1 mm from the fovea	ORL: thinner in the right nasal quadrant 0.5 mm from the fovea and the right inferior quadrant 1 mm away from the fovea			
Chorostecki et al. (2015) [121]	52 (101)	24 (46)	65.8	59.8	2.0	6.4	Spectralis OCT	Volumetric macular region	Automatic	Mean *volume* of the macular area (6 mm X 6 mm)	↑	↓	NA	NA
Müller et al. (2014) * [122]	39 (39)	33 (33)	NA	NA	NA	NA	Spectralis OCT	Volumetric macular scans	Semi-automated	Mean thickness of the macular area (6 mm X 6 mm)	NA	↔	ISOS: ↓ OPT: ↔	NA
Garcia-Martin et al. (2014) [123]	129 (129)	129 (129)	68.8	69.0	2.70	8.4	Spectralis OCT	Volumetric macular scans	Automatic	Mean thickness of the macular area (6 mm X 6 mm)	↓	↔	↔	↔
Roth et al. (2014) [124]	68 (114)	32 (63)	68.8	64.7	NA	7.2	Cirrus HD-OCT	Macular Cube region	Automatic	Mean thickness of the macular area (6 mm X 6 mm)	INL+ OPL: ↔	ONL+ PRL: ↓		NA
B. Single line scan														
Schneider et al. (2014) [125]	65	41	66.2	65.1	NA	8.9	Cirrus HD-OCT	single horizontal foveal scan	Semi-automated	Mean thickness of the macular area (4.5 mm)	↔	ONL+ PRL: ↔		NA
Lee et al. (2014) [126]	61 (56)	30 (30)	69.6	64.8	2.2	6.0	Opko OTI SD-OCT	single horizontal foveal scan	Manual	The fovea center; temporally 1 mm, 2 mm, and 3 mm, nasally 1 mm from the fovea	↔	ONL + PIS: ↔ POS + RPE: ↔		
Albrecht et al. (2012) [38]	40 (80)	35 (70)	61.2	NA	2.5	8.1	Spectralis OCT	Single horizontal foveal scan	Manual	OPL: the thickest points on nasally and temporally of the macular ONL: the central thickest point	↔	↔	NA	NA

*OCT* optical coherence tomography; *PD* Parkinson’s disease; *HC* healthy controls; *NA*, not applicable (data are not provided); ↔, no significant difference; ↓, significant decrease; ↑, significant increase; *OPL* outer plexiform layer; *ONL* outer nuclear layer; *PRL* photoreceptor layer; *IS/OS*, inner segment/outer segment junction; *RPE* retinal pigment epithelium; *PIS*, photoreceptor inner segment; *POS*, photoreceptor outer segment; *OPT* outer photoreceptor tips; *OSL*, outer segment layer; *OPR* outer segment to RPE junction; *ORL* outer retinal layer; *INL* inner nuclear layer; *ETDRS* Early Treatment Diabetic Retinopathy Study grid, contains three rings including the foveal ring (a circle of 1 mm diameter), inner ring (inner diameter of 1 mm and outer diameter of 3 mm), and outer ring (inner diameter of 3 mm and outer diameter of 6 mm)

^*^Further analysis on a subset of Albrecht et al. (2012)’ s cohort

Figure 4a shows that in the OPL, some studies demonstrated a positive Cohen’s d effect size value and significant thickening (significance indicated by *asterisks*) [117], one returned a negative Cohen’s d and significant thinning [123], while others showed no change [35, 104, 114, 116]. Of interest, Chorostecki et al. [121] reported significant OPL volume thickening. The reason for this discrepancy is unclear and is unlikely to be due to the summary metric, as thickness and volume summaries in the same cohort return similar effect sizes [117]. Only one study [25] has examined OPL thickness in iRBD participants and reported a significant thinning.

The other outer retinal layers demonstrated more consistent changes across studies with either a significant thinning or no change seen in the ONL (thinning [35, 114, 116, 117], no change [104, 122, 123]), PRL (thinning [117], no change [35, 123]), and RPE (thinning [104, 117], no change [35, 114, 116, 123]). The effect size for the PRL and RPE ranged between − 1.57 and 0.84 (including 95%CI), whereas the effect size for the ONL was larger in some studies, notably − 8.0 (95%CI − 9.9–− 7.0) in Chorostecki et al. [121] and − 1.1 (95%CI: − 1.6–− 0.6) in iRBD participants in Rascuna et al. [25].

In terms of the combined layers represented in Table 2, some studies have reported thinning of the combined ONL and PRL [124], as well as the whole outer retinal layer [115, 120, 127].

### Factors influencing variability in OCT findings

A variety of factors may contribute to differences in reported outer retina changes between PD studies. It is possible that pwPD may exhibit a particular spatial distribution of OCT changes, and averaging across a large non-homogenous region may obscure or dilute localized differences. For instance, Mailankody et al. [120] revealed a thinning of the outer retina in two specific areas (the right nasal quadrant at 0.5 mm from the fovea and the right inferior quadrant at 1 mm away from the fovea) in pwPD (60 eyes, 30 participants) compared to age-matched controls [126]. In their longitudinal study [127], the outer retinal thickness in pwPD in the temporal quadrant at 0.5 mm away from the fovea significantly decreased over 3.4 years of observation. On the other hand, the outer retinas in all individual macular sectors were thinner in 50 PD participants (100 eyes) [115]. Whether the pathology is localized to specific areas, such as the fovea, perifovea, and parafovea, or it manifests as a diffuse pattern remains unclear.

Discrepancies between studies may also arise from differences in age range, disease duration, and severity, making it difficult to compare results between studies. Also, small sample sizes (Table 2), differences in the OCT devices (e.g., swept-source OCT versus spectral-domain OCT) [128, 129], and differences in automatic retinal layer segmentation can further add to variation between studies [37, 38, 122, 125, 126, 130]. Further analyses considering relative outer and inner retinal changes in targeted retinal layers and regions (e.g., foveal, parafoveal, and superior vs inferior retina) in prodromal and established PD are warranted [25].

Segmentation of the outer retina is particularly difficult due to the retinal glial cell processes and the oblique anatomy of the macula. Lujan and colleagues [131, 132] demonstrate that standard OCT scans cause over-estimation of the ONL by ~ 50% due to obliquely oriented dendritic processes (Henle fiber layer, process of retinal glial cells) masquerading as ONL in these scans. In contrast, off-axis OCT scanning (directional OCT) enables a more accurate assessment of the ONL, OPL, and Henle fiber layers [133–135]. How accurately standard OCT scans are conducted “on-axis” is also not well documented in these studies. This is important for the outer retina as slight variations here can cause large inaccuracies in the ONL. Directional OCT imaging in pwPD or prodromal PD which enables more accurate delineation of the ONL, OPL, and usually, hidden Henle fiber layer would also be helpful for the field.

Considering the thickness changes in the inner retina are easily confounded with other neurodegenerative diseases (e.g., Alzheimer's disease [136, 137]) and ocular diseases (e.g., glaucoma [138]), outer retinal changes could reveal more disease-specific characteristics in PD. To validate the diagnostic and prognostic potential of thickness parameters in PD, further clinical studies recruiting early stage pwPD or iRBD with longitudinal follow-up observations, as well as investigation of several regions of interest within the retina are warranted.

### The underlying pathology of outer retinal alterations in Parkinson’s disease

Though it is hard to draw a definitive conclusion based on the previous findings of outer retinal changes, researchers have proposed potential mechanisms to account for retinal involvement in PD. Increases in thickness or volume have been attributed to a number of mechanisms, such as localized cell swelling associated with the initial stage of cell death [120, 127], a compensatory reaction in response to a disease-related neurodegeneration [125], and α-synuclein aggregation [117, 121]. On the other hand, tissue thinning has been attributed to degeneration secondary to impaired dopaminergic cells [121, 124], a subgroup of amacrine cells in the inner retina [85]. Another speculation of the thinning of retinal layers is a secondary change due to the α-synuclein deposition and comes from animal studies that facilitate parallel retinal tissue assessment. Our recent findings in the transgenic PD mice model (M83 expressing human A53 T variant α-synuclein) showed that there was a significantly thinner OPL and ONL, which correlated with the pathological α-synuclein accumulation in the ONL [139] Similarly, in Xu et al.’s study [73], the phosphorylated Ser129 α-synuclein accumulation was detected in the OPL of the same mouse model (M83), along with the degeneration of photoreceptor cells, including the loss of photoreceptor terminals in the OPL, and attenuated outer retinal photoreceptor function. Another finding in our toxin-induced PD mice model (MPTP) [140] has shown a significantly thinner OPL and impaired outer retinal photoreceptor function compared to the wildtype controls, indicating a toxin-mediated mechanism may be underlying the alteration of the outer retina. Other mechanisms have also been proposed as outer retinal structure-related changes, such as impaired glutamatergic pathways [127] and deprivation of docosahexaenoic acid, a material that maintains the renewal of the photoreceptor outer segment and was assumed to influence retinal degenerative disorders [119].

## Conclusion

Color vision deficiency is commonly observed among those with PD and prodromal PD, and in some cases, color vision deficiency can be detected even a decade before the onset of motor symptoms. Defects have been reported in the red–green axis as well as the blue–yellow axis. The association between color vision loss and the disease severity of PD remains uncertain, requiring longitudinal observation in a larger cohort with consistent methodologies. Given scalability issues with current color vision tests, the development of widely implementable color vision tests for population screening would be very useful. Whether vision assessments should be included in future PD rating scales warrants future consideration to assist in diagnosis, progression, and severity grading. OCT imaging is widespread in the community, affording an opportunity for population-based screening of PD. The outer retinal layers observed by OCT include the photoreceptor and retinal pigment epithelium cells, and changes in outer retinal thickness may be useful as a more disease-specific measure in pwPD than generalized inner retinal thinning. Further work is needed to pinpoint the optimal OCT imaging approach and location to return the most useful diagnostic and monitoring metrics.

## Data Availability

All original sources are cited in the manuscript. Data extracted and analyzed in this review are available from the corresponding author upon request.
